# Src-family Protein Tyrosine Kinases: A promising target for treating Cardiovascular Diseases

**DOI:** 10.7150/ijms.49241

**Published:** 2021-01-14

**Authors:** Yuhong Zhai, Jun Yang, Jing Zhang, Jian Yang, Qi Li, Tao Zheng

**Affiliations:** 1 Department of Cardiology, The First College of Clinical Medical Science, China Three Gorges University, Yichang 443000, China.; 2 Institute of Cardiovascular Diseases, China Three Gorges University, Yichang 443000, China.; 3 Central Laboratory, Yichang Central People's Hospital, Yichang 443000, China.

**Keywords:** Src-family protein tyrosine kinases, hypertension, coronary heart disease, ischemic heart disease, myocardial ischemia reperfusion injury

## Abstract

The Src-family protein tyrosine kinases (SFKs), a subfamily of non-receptor tyrosine kinases, are ubiquitously expressed in various cell types. Numerous studies have suggested that SFKs are related to signal transduction in major cardiac physiological and pathological processes, it is the activity of SFKs that is connected with the maintenance of cardiovascular homeostasis. Upon stimulation of various injury factors or stress, the phosphorylation state of SFKs is changed, which has been found to modulate different cardiac pathological conditions, such as hypertension, coronary heart disease, ischemic heart disease, myocardial ischemia-reperfusion injury, arrhythmia and cardiomyopathy via regulating cell growth, differentiation, movement and function, electrophysiologic signals. This review summarizes the basic information about SFKs, updates its role in the different processes underlying the development of multiple cardiovascular diseases (CVDs), and highlights their potential role as disease biomarkers and therapeutic targets, which would help understand the pathophysiology of CVDs and promote the further potential clinical adhibition.

## Introduction

The epidemiological tendency in the 20^th^ century was accompanied by an increase in noncommunicable diseases, of which cardiovascular diseases (CVDs) are now the leading cause of mortality and morbidity worldwide 1. CVDs are the most common leading cause of death globally, representing 31% of all global deaths. In 2017, it was about 17.8 million people that died of CVDs worldwide. In China, the steadily rising incidence and prevalence of CVDs is likely to continue some time, particularly for coronary artery disease, stroke, and heart failure. Multiple factors are contributing to the epidemic of CVDs, including the rapid aging of the population, improved survival rate from other illnesses, progressive urbanization, increased calorie consumption, decreased physical activity, mental stress, and air pollution 2-4. Therefore, it is high time to pay more attention and social resource to patients with CVDs and high-risk groups.

The etiology of CVDs is complex, and there are multiple pathogenic factors and pathogenesis. Several studies demonstrated that Src-family protein tyrosine kinases (SFKs), the largest subfamily of non-receptor intracellular kinase, express not only on haematopoietic lineage cells such as platelet, monocytes/macrophages, also highly on vascular smooth muscle (VSM), endothelial cells and myocytes^5^-^9^. They play a variety of important regulatory roles in many different cellular signaling pathways through interacting with a variety of substrates *in vivo* including platelet-derived growth factor, epidermal growth factor receptor, p130Cas, cortactin and so on^10^. In a subsequent study, it was found that these bioactive factors was bound up with the normal vascular function and disease progression 11. In this review, one of the objectives focuses on structure and function of SFKs as well as the role of the various members in the initiation, progression and development of different forms of CVDs, another one pays attention to how to use SFKs to provide novel ideas for the diagnosis and treatment of cardiovascular diseases.

## Structure and biological characteristics of SFKs

**Figure 1:**
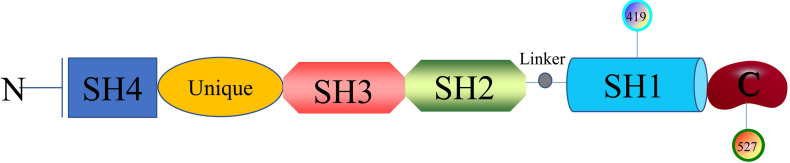
Structure of SFKs.

The researches of SFKs originated from Peyton Rous's study about Rous sarcoma virus in birds solid tumours in 1911, avian cancer-causing oncogene could express protein v-src and cellular physiological proto-oncogenes gene could encode Src 12. Next, it was found that there are at least nine members in the large SFKs in humans, including Src, Lck, Hck, Blk, Fyn, Lyn, Fgr, Yes, and Yrk. Also, there are differences in the distribution of these members in the tissues. Src, Fyn, as well as Yes are ubiquitously expressed in most cell lines 13. Lyn and Fgr are predominantly in hematopoietic system 14, Lck is mainly in T lymphocytes and natural killer cells 15, Hck is confined to myeloid cells and B-lymphocyte lineages 16, Blk is in B cells 17, and Yrk is majorly in monocytes 18. SFKs are 52-62 kDa proteins with highly homologous in structure (Figure 1), from the N- to C-terminus, members of the family exhibit a 14-carbon myristoyl group and sometimes a palmitoylation site attached to an SH4 domain, followed by a unique domain, an SH3 domain directing specific association with proline-rich motifs which adopt a left-handed helical conformation, an SH2 domain providing interaction with phosphotyrosine motifs, an SH2-kinase linker and an SH1 tyrosine kinase domains, last a short C-terminal regulatory tail^19^. The Myristoylation, an irreversible modification catalyzed by the N-myristoyl transferases (NMTs), and the palmitoylation that is a dynamic and reversible process mediated by Acyltransferases are required for the membrane anchor and SFKs activation 20. The SH3 domain always binds to specific proline-rich peptides of partner proteins; meanwhile, a SH2 domain connects with specific tyrosine phosphorylation sites to mediate protein-protein interactions 21. A SH1 domain contains the Tyrosine kinase catalysis site, an ATP-binding pocket and the tyrosine-specific protein kinase activity 22. Under normal physiological conditions, the domain SH2 links up with phosphorylated tyrosine residue 527 in the C-terminal tail to maintain SFKs in a closed, inactive state, and the interaction between SH3 with the linker helps to stabilise the inactive conformation. Tyrosine 416, an autophosphorylation site in the protein-tyrosine kinase domain, can autophosphorylate to stabilize the open, active state of SFKs after phosphorylated tyrosine residue 527 is dephosphorylated or external ligands from other proteins bind to SH2 or SH3 domain 20, 23. The transition of SFKs from active forms to inactive forms is dynamic, and during which it is upstream regulators that release the structural constraints to finish this 14. So, it is of great significance to monitor or intervene the states of SFKs.

SFKs that have no extracellular portion and no integral plasma membrane-spanning domain are activated by cell-surface specific receptors, such as the cytokine receptor, Tyrosine kinase receptors, growth factor receptor, G-protein-coupled receptors, T cell receptor, and B cell receptor, activated SFKs recruit and phosphorylate multiple downstream signaling factors by catalyzing the transfer of γ-phosphates from ATP molecules to tyrosine residues of target proteins, thereby transmitting extracellular signals to downstream cellular components, then SFKs finally control a series of signal networks that regulate metabolism, viability, proliferation, differentiation, and migration in many different cell line^24^, ^25^. For instance, SFKs activated by lipopolysaccharide (LPS) could phosphorylate Toll-like receptor 4 and then dissociate MyD88 and Mal/Tirap to suppressing inflammatory responses 26. In all lymphoid cells expressing CD45, an inhibitor of CD45 led to abnormal SFKs signaling, resulting in a G2/M cell cycles arrest and apoptotic cell death 27. In other organizations, the total levels and the activation levels of specific SFK members were also important for regulating apoptotic activity 28. Flora et al. reported that down-regulation of SFKs activity negatively regulated platelet functions, thrombosis, and haemostasis 29. All in all, SFKs are the key to regulating the life activities of the body of every aspect.

## SFKs and cardiovascular diseases

### SFKs and hypertension

Hypertension is a major preventable risk factor and all-cause mortality of CVDs worldwide, which can cause much damage to vital organs, namely the cardiovascular, neurological, and renal systems 30. The pathogenesis of hypertension is related to left ventricular hypertrophy, overactivation of the renin-angiotensin-aldosterone system and sympathetic nervous system, dysfunction of vascular endothelium and vascular smooth muscles, aberrant angiogenesis and remodeling in small arteries 31.

Blood pressure is a complex network regulated by a series of physiological pathways. It is reported that the C-terminal Src kinase (Csk) works as a negative regulator of SFKs and a core modulator of blood pressure physiologically via inhibiting the adrenal gland to synthesize more aldosterone, regulating the contraction, growth and migration of VSMs, changing in the blood volume and sodium absorption, ultimately affects blood pressure levels 32, 33. And it has been confirmed that gene silencing and Haploinsufficiency of Csk could increase blood pressure via up-regulating the active form of Src 34. That increased angiotensin II promotes the contraction of smooth muscle cells is another cause of hypertension with the deep research 35. Angiotensin II not only regulates the phosphorylation of many tyrosine kinases, such as Src, Janus family kinases, focal adhesion kinase, Pyk2, p130Cas and phosphatidylinositol 3-kinase via angiotensin II type 1 receptors and also augments angiotensin II-induced activation of Src 36. Qin et al. compared the hypertension model group with SFKs inhibitor SU6656 group, then demonstrated that inhibition of SFKs lowered the level of blood pressure in angiotensin II-treated mice without affecting the normal mice, in that SFKs inhibitors could reduce the phosphorylation of myosin-light-chain in human coronary artery smooth muscle cells 37. It is reasonable that SFKs directly regulate vascular contractile machinery to affect blood pressure. Early study has shown that some members of SFKs could closely associate with their substrate, N-methyl-Daspartate receptors (NMDARs), via indirect and direct binding mechanisms 38. Whereafter, Qiao et al. also found that the activity of NMDARs and the level of Src protein in the paraventricular nucleus of spontaneously hypertensive rats was significantly higher than that in normotensive rats. Inhibition of Src kinase activity normalized the burst of evoked NMDARs-mediated higher excitatory postsynaptic currents and weakened the rostral ventrolateral medulla currents induced by NMDARs, but did not affect normal rats 39. These data suggest that SFKs which are vital for the normal can intervene in a variety of pathologic mechanisms and different types of hypertension as a critical mediator.

### SFKs and coronary heart disease

Coronary artery disease (CAD) is a serious cause of death worldwide with data from the “Summary of the 2018 Report on Cardiovascular Diseases in China” showed that the number of individuals with CAD has reached 11 million 40. CAD mainly includes arterial sclerosis of the coronary arteries, angina pectoris, myocardial infarction as well as consequent chronic heart failure 41. It is widely accepted that the occurrence and development of CAD are related to many factors and SFKs participate in the process.

Atherosclerosis is a progressive disease characterized by a gradually developing atherosclerotic plaque containing lipid and immune-cell deposition in parts of the artery accompanied by smooth muscle cell and fibrous matrix proliferation 42, 43. SFKs can regulate foam cell formation and proinflammatory cytokine expression, efferocytosis, lesion development and instability in a mouse model of atherosclerosis 6. That means SFKs have a key role, acting as a common basis for the physiological and pathological changes throughout atherosclerosis initiation and development 44. During early atherosclerosis, Marcovecchio et al. demonstrated that one of SFKs regulated the role of scavenger receptor CD36-mediated non-classical monocytes patrolling through DAP12 junction proteins to play a protective role in early atherosclerosis 45. However, various researches have shown that the kinase Lyn activated by binding of oxidized low-density lipoprotein (oxLDL) to scavenger receptor CD36 could initiate the cascade reaction to accelerate foam cell formation induced by oxLDL which was a hallmark of atherosclerosis^46^, ^47^. Harrison et al. used *in vitro* adhesion assays and single SFK knockout mice crossed with the ApoE^-/-^ model of atherosclerosis, SFK signal regulated platelet-dependent leucocyte accumulation of monocytes, as knocking out Fgr or Lyn gene inhibited the recruitment of monocytes in vascular inflammation model 48. That indicated SFKs signaling was not redundant in Atherosclerosis and there are at least two regulatory pathways related with SFKs. An individual number of SFKs would represent targets for therapeutic intervention. These studies show that SFKs are involved in the regulation of inflammation, a particular hallmark of atherosclerosis.

Another main presentation of CAD may be ischemic heart disease (IHD), it is caused by coronary atherosclerosis and/or thrombosis and leads to injury or death of cardiomyocytes due to ischemia and/or hypoxia. Clinically, the broad sense of IHD can be divided into angina pectoris (AP), myocardial infarction (MI), heart failure (HF), acute coronary syndromes (ACS), sudden cardiac death and so on 49-51. There is no direct evidence to support the correlation between IHD and SFKs. However, some studies have proved that SFKs play an important role in the pathological process of IHD indirectly. SFKs are widely found in platelets which are an essential component of the thrombus formation following plaque rupturing and playing a key role in activating and transducing platelet signal pathways, at last leading to ACS 52, 53. Src, Lyn and Fyn are the three most abundant SFKs in human and mouse platelets, Src and Fyn play a positive role in regulating platelet activation, whereas Lyn is both a positive and negative regulator of activation 53. Speich et al. used SFKs inhibitors SU6656 and PP2 to pretreat platelets, restraining the upstream site of spleen tyrosine kinase (Syk) could substantially recede shear-induced phosphorylation at Syk to decrease shear-induced platelet aggregation 54. And another study has found that a potent inhibitor PP2 had a suppressive influence on the release of platelet-derived brain-derived neurotrophic factor which was beneficial for both diabetic neuropathies and cardiovascular injury 55. SFKs interact well with diverse platelet surface receptors like GPVI, PECAM, PEAR1, and CD148. In turn, the genetic variants of platelet receptors can influence activation of SFKs and then platelet, the platelet's hyperactivation which is essential for thrombus growth and stability may result in MI and cardiovascular death 56. There are many factors can influence the interaction between SFKs and platelet, but unfortunately, there is not enough evidence to clarify its relationship. In general, SFKs indirectly play a role to intervene in the pathological process of the IHD by influencing the function of platelet which has been linked to acute myocardial infarction, unstable angina, and stroke. HF mostly caused by potential myocardial disease is defined as the inability of the heart to supply the peripheral tissues with the required amount of blood and oxygen to meet their metabolic demands and is usually described by left ventricular ejection fraction 57. During the transition from compensated pressure-overload hypertrophy to decompensated congestive heart failure, Takeishi et al. found that chronic pressure-overload and acute mechanical stretch activated Src in normal hearts, which played a role as a novel signal transduction site to protect the heart from excess cardiac hypertrophy in response to additional stimuli like acute pressure-overload 58. The hyperactivation of neurohormonal systems especially β-adrenergic receptors (βARs) signaling abnormalities plays a pivotal role in HF pathogenesis 59. And activated βARs directly lead to the phosphorylation of signal transducer and activator of transcription 3 (Stat3) which integrates multiple signaling pathways in the development of cardiac hypertrophy and eventual heart failure in cardiomyocytes 60. Zhang et al. investigated the molecular mechanism underlying βAR-mediated Stat3 activation and then proved that Src, Yes and Fyn, the main SFKs in MEF cells were critical mediators for βAR-initiated Stat3 phosphorylation to maintain the physiological function of the heart 61. What if SFKs phosphorylate or influence the pathological process of heart failure induced by βARs? So, a lot of researches are needed to clarify its role.

### SFKs and myocardial ischemia reperfusion injury

Myocardial ischemia reperfusion injury (MIRI) is the main complication of reperfusion therapy after acute MI. thrombolytic drugs, percutaneous coronary intervention and coronary artery bypass graft surgery, the first choice for rapid recovery of cardiac blood supply and reduction of myocardial tissue injury, are the primary reasons for MIRI 62. It is mainly manifested in myocardial necrosis, stunning, poor reperfusion, no-reflow and reperfusion arrhythmias, the pathological mechanisms of MIRI include oxygen free radical formation, calcium overload, neutrophil-mediated myocardial and endothelial injury, progressive decline in microvascular flow to the reperfusion myocardium and depletion of high-energy phosphate stores 63. Hence, looking for a target to reduce pathological injury should be the main goal in the treatment of myocardial ischemia.

The first literature about the expression of SFKs in cardiomyocytes has shown that adult rabbit cardiomyocytes stably expressed following 7 members in SFKs: Fyn, Fgr, Yes, Src, Lyn, Lck, and Blk. Moreover, the activation of Src and Lck was associated with ischemic preconditioning in conscious rabbits. Among them, the Change of Src is very obvious after ischemic preconditioning 64. Then, more and more researchers concentrated on the relationship between Src and MIRI In future experiments. At first, it has been confirmed that hypoxia/reoxygenation caused enhancement of autophosphorylation of cell surface-associated SFKs, as Src and Fyn in cardiac myocytes from the neonatal rats at an early stage 65. And, Li et al. pretreated SD rats and H2C9 cells with pNaKtide which can bound and reduced the activities of Src and its effectors forcefully, following established reperfusion injury models *in vivo* and *in vitro*. Emerging data suggested that PNaKide inhibited the phosphorylation of Src and its downstream effect factor extracellular signal-related kinase 1/2 in a dose-dependent manner to reduce the reactive oxygen species accumulation as well as apoptosis. However, mitochondrial SFKs including Lyn, Src, Fyn, and Fgr have been found in mitochondrial compartments in recent years. Interestingly mitochondrial Src tyrosine kinase is inhibited by H/R from rat hearts. In other words, Src tyrosine kinase was inactivated in MIRI 66. phosphorylated Src expression in the mitochondria decreased with an increased mitochondrial ROS level after H/R treatment through JNK/Sab/Src/ROS pathway^67^. And, the activation of Src concentrates on various components of the reperfusion injury salvage kinase pathway that has been proved as a potential target for protecting the heart from ischemic/reperfusion during the period of ischemic preconditioning 68. So, the mechanism of SFKs is intricate, whether they are present in other organelles beyond these two structures,the specific effect of SFKs on the MRI model is remaining to be explored. Taken together, these studies reveal that Src represents a key intermediate and new therapeutic target in the pathophysiology of MIRI. Combined with our early research, it is radioprotective 105 kDa protein (RP105) highly expressed on B-cells, a member of the TLRs's family, that attenuates myocardial inflammation, apoptosis, autophagy response and decreases infarct size as the inhibitor of the TLR4 signaling pathways in the myocyte in MIRI 69, 70. In the study of immune cells, someone demonstrated that RP105 ligation induced Lyn activation, recruited CD19 into lipid rafts, and then induced Lyn phosphorylating CD19 to recruit Vav, the Lyn/CD19/Vav complex is essential for JNK activation via TLR4-independent pathway when LPS activates B cells 71, another found that RP105 ligation inhibited the interferon-α signaling through the Lyn-PI3K-BTK signal axis which was also different from TLR4 pathway 72. So, it is worth pondering whether RP105 or some damage-associated molecular patterns can mediate SFKs-induced other signal pathways in MIRI.

### SFKs and arrhythmia

Arrhythmia is the occurrence of abnormal frequency, rhythm and conduction velocity during normal myocardial activation or beating of the heart myocardium. There are many types of arrhythmias, and their severity is related to the occurrence of structural lesions in the myocardium 73, 74. Hence, malignant arrhythmia is the main topic.

Arrhythmias often occur in a variety of structural heart diseases, other systemic diseases, diet, exercise as well as drugs both are risk factors for arrhythmia 75. During the first-phase clinical study of the combination of the SFKs inhibitor dasatinib and all-trans retinoic acid in patients with acute myeloid neoplasms. Redner et al. found that dasatinib caused the patient, with a history of coronary artery disease and coronary artery bypass grafting, to develop arrhythmia grade 3 prolongation of QTc, and that is one side effect of dasatinib 76. Thus, it is reasonable to suspect that there is a connection between SFKs and arrhythmia. Lin et al. compared the effects of Src, Fyn, and Yes on hyperpolarizing-activated cyclic nucleotide-gated 4 pacemaker channels with its mutant D553N, found in a patient associated with cardiac arrhythmias. They showed in this report, actived SFKs: Src, Fyn and Yes, improved the gating properties of D553N 77. These three SFKs were able to restore the surface expression of D553N for normal current expression through enhancing tyrosine phosphorylation in the heart. Several subsequent studies have verified that SFKs activity is potential in connection with cardiac pacemaker activity 78. In another research, Src inhibition reduced the internalization and degradation of connexin 43, a main component of gap links in the whole heart, to improve conduction velocity and lessen arrhythmia inducibility following MI 79, 80. Here, it does not systematically discuss the SFKs specific effect on the pathogenesis of arrhythmia, representing our future research endeavors. Therefore, it is significant to discover the effective endogenous regulatory mechanism of SFKs to correct cardiac arrhythmias.

### SFKs and other heart diseases

Viral myocarditis caused by group B coxsackieviral (CVB) infection got relieved following gene-targeted knockout of Lck in mice, Lck and other SFKs could principally regulate CVB3 replication in T cells, dendritic cells, B cells and macrophages 81. Tracing its downstream signal factors, Opavsky et al. found that BERK-1/2, a signal protein downstream of Lck, could be activated by Lck and other SFKs to finish the efficient CVB3 replication in both T cells and cardiac myocytes infected. The identification of SFKs, especially Lck as an essential regulator of CVB3 replication may allow the design of drugs that specifically interferes with the replication and persistence *in vivo* of this virus, avoiding the development of viral myocarditis 82. And in research of hypertrophic cardiomyopathy, it has been demonstrated that Fyn and NADPH oxidase 4 (NOX4) are co-located in the perinuclear region of cardiomyocytes, the N-terminal unique domain of Fyn phosphorylated tyrosine 566 in the C-terminus of NOX4 through their interaction and negatively regulated NOX4-induced exacerbation of pathological myocardial hypertrophy 83. In addition, some studies also found that there is a close relationship between SFKs and the development of cardiac valvular disease. Hutcheson et al. found that 5-HT_2B_ antagonism changed the function and spatial location of Src and physically restricts it to preventing downstream signaling via non-canonical transforming growth factor-β1 (TGF-β1)-p38 MAPK signaling rather than the phosphorylation of Src, that could finally slow down TGF-β1-induced myofibroblast activation of quiescent aortic valve interstitial cells, a differentiation process implicated in calcific aortic valve disease 84. In a word, according to the above results, it is not difficult to find that SFKs also play an important role in other cardiovascular diseases.

## SFKs-related possible clinical application

In conclusion, the numbers of SFKs are involved in the development of CVDs, how to make use of discoveries to provide convenience for the diagnosis and treatment of clinical CVDs is what most researchers care about. Alexandar V et al. referred to the literature related to CVDs to construct a network of protein interactomes, then identified Src as one of the potential candidate markers, further validated it with existing markers in volunteers via ELISA. The results suggested that the level of Src in the serum of the patients changed significantly 85. These support the utility of Src as candidate markers for the diagnosis of CVDs. Analogously, Yan er al. downloaded the comprehensive database of gene expression of the sample population to analyze the correlation between genes and CVDs, the analysis of overlapping genes showed that Lck is one of the top three genes in a protein-protein interaction network. Nevertheless, related diagnostic techniques have not become popular without a large number of clinical trial data 86. The accuracy, feasibility and effectiveness of using SFKs in the diagnosis of CVDs need more clinical trials to be tested. In early study, virtual experimental methodologies as GRID and Sybyl docking algorithms indicating that flavonoids which can reduce the risk of CVDs through anti-inflammatory, anti-coagulant and anti-platelet actions, interface with kinase catalytic site residues including Lyn, Fyn and Hck through potential hydrogen bond, finally inhibit the activity of those SFKs 87. Next, Vallance et al. briefly analyzed the current knowledge on the impact of the flavonoids on the modulation of the platelet function, naturally occurring flavonoids such as nobiletin, tangeretin, quercetin, apigenin, luteolin, rhoifolin as well as synthetic flavonoids which had improved bioavailability including multiple synthetic 2,3-diphenyl-4H-pyrido(1,2-α)pyrimidin-4-one flavonoids, F-1 both could inhibit platelet activation via multiple mechanisms. However, at present, there is a lack of standardized use of flavonoids in clinical treatment guidelines 88. The problems relating to the bioavailability, stability and multiple cellular targets of compounds remain to be solved. It is essential to develop and modify flavonoids to make them more effective lead compounds for drug design.

## Discussion

SFKs exist widely, and we mostly generalized the function and potential pathway of SFKs in different CVDs. Based on extensively studies about the pathogenesis of those diseases, we think the potential pathophysiological mechanism related to SFKs has not been fully understood. At first, most of the above researches define SFKs as the plasma membrane, interestingly, SFKs are also found to localize to organelle membranes including Golgi membranes and lysosomal membranes and mitochondrial membranes 89. The intracellular location is dynamic, such as the enrichment of activated Fyn in multivesicular bodies led to a defect in cell differentiation in a neuroblastoma cell line 90. Next, the study on the pathological mechanism of SFKs in the above-mentioned diseases is not in-depth enough, SFKs can phosphorylate multiple sites of relative proteins in mammalian cells 91, or the presence of bi-phosphorylated SFKs was found in triple-negative breast cancer model 92. The phosphorylation sites of key upstream targets and receptor proteins related to CVDs need to be more accurate for a deep research from many aspects and levels. Third, the members and functions of SFKs are complex and diverse, for example, Src was expressed in all malignant skin tumors 93, Fyn assembled in response to adult ischemic brain injury 94. However, few studies have examined the relative effect of specific SFKs in CVDs with using specific SFKs inhibitors. Meanwhile, the drugs developed with the target of some SFKs for CVDs are limited, except for large common number of SFKs inhibitors. Vast computational studies based on receptor and/or ligand virtual screening, docking, and molecular modeling proved to be a powerful tool for identifying new SFKs inhibitors and have a promising application 95. Further development of drugs with high efficiency, specificity and few unwanted and dangerous side effects along with medical science rapid growth would provide a greater opportunity for treatment of CVDs. Finally, the role of SFKs in another CVDs including endocardiosis, congenital heart disease, rheumatic heart disease… need to be clear, collecting a large amount of clinical evidences and establishing relevant experimental models will be helpful to broaden the scope of SFKs. And, there is a lack of large samples, multicenter clinical observation, literature, suggesting it is of great significance to make further research to expose the neglected role of SFKs in cardiovascular fields.

## Conclusion

To sum up, SFKs can regulate the process of inflammation, platelet activation, blood volume, various cellular functions and other factors related to the occurrence and development of CVDs from the increasing research. As the important molecules, these kinases can be considered important targets to regulate extracellular signal transduction in different human diseases. Therefore, it is of great clinical significance to study the mechanism and role of SFKs in CVDs, to reveal the relationship and find effective intervention measures to prevent the occurrence and progression of cardiovascular disease. Future studies on SFKs to provide the theoretical foundation for diagnosis, treatment and prevention of CVDs in the clinic are of great concern.

## Abbreviations

SFKs: Src-family protein tyrosine kinases
CVDs: cardiovascular diseases
VSM: vascular smooth muscle
NMTs: N-myristoyl transferases
LPS: lipopolysaccharide
Csk: C-terminal Src kinase
NMDARs: N-methyl-Daspartate receptors
CAD: coronary artery disease
oxLDL: oxidized low-density lipoprotein
IHD: ischemic heart disease
AP: angina pectoris
MI: myocardial infarction
HF: heart failure
ACS: acute coronary syndromes
Syk: spleen tyrosine kinase
βARs: β-adrenergic receptors
Stat3: signal transducer and activator of transcription 3
MIRI: Myocardial ischemia reperfusion injury
RP105: radioprotective 105 kDa protein
CVB: B coxsackieviruses
NOX4: NADPH oxidase 4
TGF-β1: transforming growth factor-β1
